# Host-Dependent Endophytes as a Resilient Biological Reservoir: Niche-Specific Expansion and Holobiont Reassembly in Grapevine

**DOI:** 10.3390/microorganisms14081780

**Published:** 2026-08-12

**Authors:** Jing-Xiu Tang, Yu-Tao Wang, Hong-Yan Hu, Jia-Xin Zhou, Rui-Yu Yang, Qiu-Yue Zhang, Hao Sun, Xiao-Xia Pan, Ming-Zhi Yang

**Affiliations:** 1 Yunnan Key Laboratory for Plateau Mountain Ecology and Restoration of Degraded Environments & School of Ecology and Environmental Sciences, Yunnan University, Kunming 650504, China; tangjingxiu722@163.com (J.-X.T.); wyt228657@163.com (Y.-T.W.); hucarrot1215@163.com (H.-Y.H.); zhoujiaxin8818@163.com (J.-X.Z.); yry13698788764@163.com (R.-Y.Y.); zhangqiuyue0617@163.com (Q.-Y.Z.); sunhaozjj@126.com (H.S.); 2 Institute of International Rivers and Ecological Security, Yunnan University, Kunming 650504, China; 3 School of Chemistry and Environment, Yunnan Minzu University, Kunming 650504, China; pan901805@126.com

**Keywords:** host-dependent endophytes (HDEs), niche-specific redistribution, grapevine, habitat transition, core microbiome

## Abstract

Plants and their endophytic microbiota form a functional holobiont, wherein host-dependent endophytes (HDEs) are proposed to serve as persistent microbial components associated with host continuity. Using long-term in vitro grapevine plantlets, we investigated HDE redistribution and reassembly across successive agar-based subcultures and during the transition to soil-based cultivation. Results showed that the initial tissue niche (shoot-tip versus root-base) imprinted a persistent compositional signature on HDE communities, with distinct cultivar-dependent effects. Numerous microbial amplicon sequence variants (ASVs) remained shared across agar-based and soil-based habitats, demonstrating high persistence of HDE-associated lineages. Bacterial communities exhibited marked plasticity, with plant-associated bacterial lineages contributing substantially to soil community assembly. Notably, 204 root-enriched bacterial ASVs, comprising 10.8–14.3% of the soil community, were recovered, revealing a robust “Root-Specific Legacy”. Bacterial HDEs showed higher redistribution potential (43.3%), whereas fungal transfer was significantly lower (10.4%). Functional profiling suggested habitat-associated functional patterns, with the anaerobic phenotype enriched in soil and predicted enhancements in membrane transport, nucleotide metabolism, glycan biosynthesis, and biosynthesis of secondary metabolites in roots. *Cladosporium* and *Fusarium* remained dominant in the fungal core taxa, and habitat transition altered their predicted trophic profiles. Our findings establish HDEs as a resilient biological reservoir capable of niche-specific expansion, contributing to the ecological reassembly of the plant holobiont.

## 1. Introduction

Plants and their endophytic microbiota form an intimately associated functional entity, termed the holobiont [1,2]. Within this system, endophytes influence plant adaptation by regulating key physiological processes, including metabolism, nutrient acquisition, and stress responses [3,4,5,6]. The mode of microbial transmission governs host-microbe evolutionary relationships [7,8], with endophytes in certain plants classified as horizontally transmitted endophytes or vertically transmitted endophytes [9]. In contrast to horizontally acquired endophytes [10,11], which are typically recruited from the environment, vertically transmitted endophytes are directly transferred to the next generation via seeds or vegetative propagules [12,13,14,15], such as *Pantoea* [16,17] and other microbial taxa that have been validated as vertically inherited symbionts. This vertical transmission ensures the consistent inheritance of specific symbiotic lineages and enriches for host-dependent endophytes (HDEs) [18]. HDEs show strong tissue specificity and, due to their reliance on host conditions, are often difficult to culture in vitro [19]. This close association functions analogously to a heritable trait of the plant [20].

Plantlets maintained under long-term in vitro culture represent a physically isolated system that limits external microbial input [21,22]. As a vegetatively propagated perennial crop, the grapevine provides an appropriate system for examining microbial persistence across repeated subcultures [23]. Within this isolated system, stable microbial communities are enriched in host-associated lineages [24]. However, the plant interior is not homogeneous [25]. Different tissue niches, with distinct physicochemical microenvironments, lead to spatial segregation of HDEs [26,27,28]. Key questions remain about whether this spatial pattern is maintained across multiple subculture generations and how the community reassembles after the in vitro cultured plantlets are transferred to a soil cultivation system. Addressing these questions is crucial for understanding the stability and host control of plant-microbiota symbioses under microbe-limited conditions.

In this study, we analyzed the spatial distribution and persistence of vegetatively transmitted endophytes in long-term subcultured grapevine. After transferring plantlets to sterile soil, we assessed microbiota stability and community redistribution, quantified the roles of host genotype and tissue niche in community reassembly, and examined endophytic dispersal. This work advances the understanding of host-associated microbial continuity in plants and may inform microbiome-based strategies for perennial crop improvement.

## 2. Materials and Methods

### 2.1. Plant Materials and Experimental Design for Habitat Transition

Two long-term subcultured grapevine cultivars were used in this study: Cabernet Sauvignon (*Vitis vinifera*) (C) and Rose Honey (*V. vinifera* × *V. labrusca*) (R). The original plant materials were obtained from the vineyard at Chenggong Campus, Yunnan University (102.85° E, 24.83° N), Kunming, China. The plantlets had been maintained for over 10 generations. All plantlets were routinely cultured on Murashige and Skoog (MS) solid medium supplemented with 0.4 mg/L indole-3-butyric acid (IBA), 0.1 mg/L 6-benzylaminopurine (6-BA), 30 g/L sucrose, and 7.6 g/L agar, with the pH adjusted to 5.8 ± 0.1. The cultures were incubated at 25 ± 2 °C under a 16 h light/8 h dark photoperiod and subcultured every 28 days.

**Figure 1:**
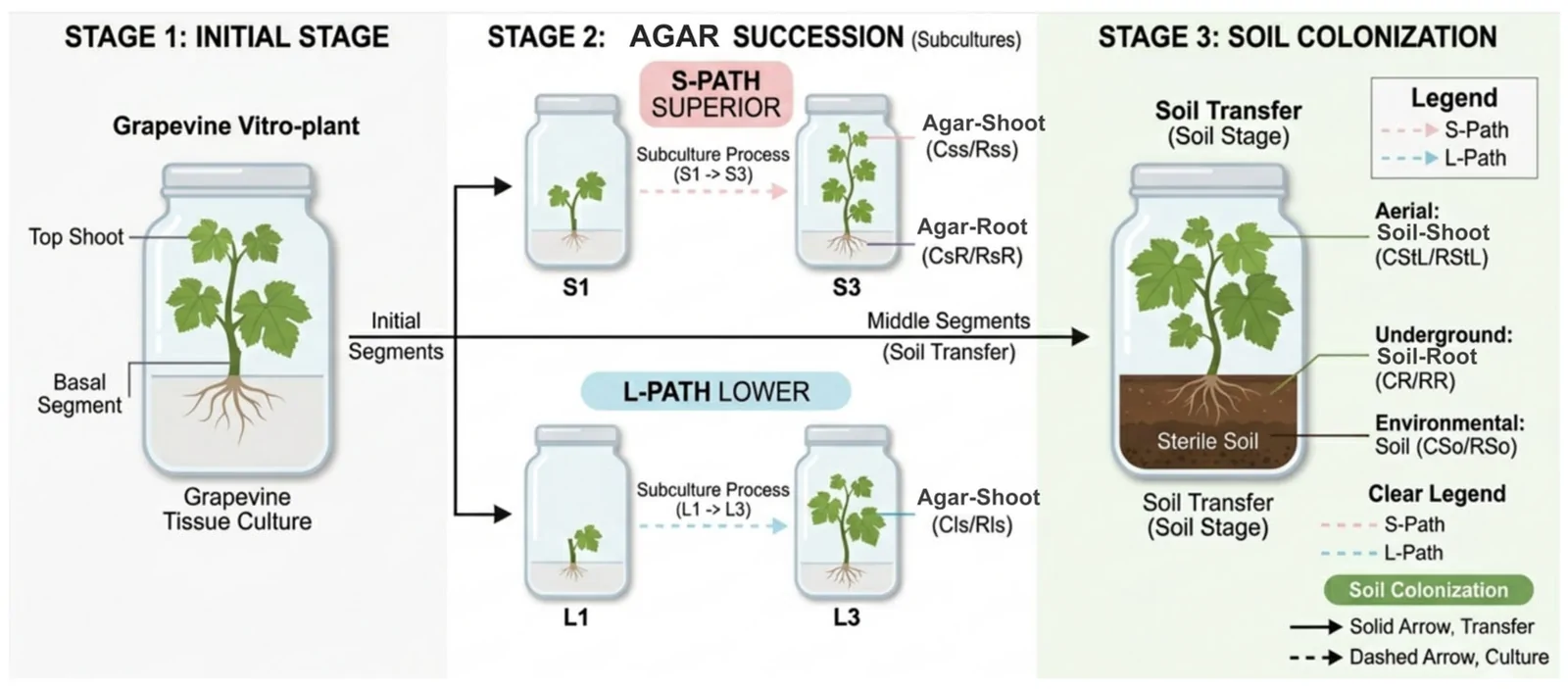
Experimental design flowchart. Two subculture pathways (S-pathway from shoot tips, L-pathway from basal stem segments) were established from long-term in vitro grapevine plantlets. Tissues from the third generation (S3 and L3) were sampled (agar-Shoot and agar-Root). Concurrently, middle stem segments were subcultured and transplanted into sterilized soil, followed by sampling of stem-leaf, root, and rhizosphere soil (soil–Shoot, soil–Root, and Soil).

To investigate the spatial inheritance of host-dependent endophytes, two distinct subculture pathways were established from the original plantlets: the Shoot-tip pathway (S-pathway) and the Lateral-basal pathway (L-pathway). The S-pathway was initiated from apical shoot tips (designated S1) and the L-pathway from basal stem segments containing lateral buds (designated L1). Shoot tips from S1 plantlets were subcultured to generate the S2 generation, and basal segments from L1 plantlets to generate the L2 generation. This targeted subculture was repeated to produce the third generation (S3 and L3). All the subcultures above were carried out in an agar-based culture system. Stem-leaf tissues (agar-Shoot) from S3 plantlets were labeled Css (for Cabernet Sauvignon) and Rss (for Rose Honey), and from L3 plantlets as Cls and Rls, respectively. Root tissues (agar-Root) from all S3 and L3 plantlets were collectively sampled and labeled CsR/RsR. Tissues from three biological replicates (*n* = 3; one plantlet each) were subjected to high-throughput sequencing. In parallel, the remaining middle stem segments (following the removal of S1 and L1 explants) were subjected to consecutive routine subculturing. Uniform plantlets from this line were aseptically transplanted into sterilized soil (by autoclaving three times at 121 °C for 20 min each time, with a 2-day interval) for a soil-based culture system [29], in a controlled growth chamber. After a complete growth cycle, samples were collected, including stem-leaf tissues (soil–Shoot: CStL/RStL), roots (soil–Root: CR/RR), and the associated rhizosphere soil (Soil: CSo/RSo), each in three biological replicates. All samples were immediately snap-frozen in liquid nitrogen and stored at −80 °C until analysis. The complete experimental workflow, including the two subculture pathways and soil transfer, is illustrated in Figure 1.

### 2.2. DNA Extraction, PCR Amplification, and Sequencing

Total genomic DNA was extracted from 500 mg of tissue or 250 mg of soil samples using the Bacterial Genomic DNA Extraction Kit DP302-02 (TIANGEN; Beijing, China). DNA concentration was accurately quantified using a Qubit fluorometer (Waltham, MA, USA). For the characterization of bacteria in the samples, the V3–V4 hypervariable region of the 16S rRNA gene was amplified with primers 341F (5′-CCTACGGGNGGCWGCAG-3′) and 805R (5′-GACTACHVGGGTATCTAATCC-3′) [30]. For fungi, the ITS region was amplified using primers ITS1FI2 (5′-GTGARTCATCGAATCTTTG-3′) and ITS2 (5′-TCCTCCGCTTATTGATATGC-3′) [31]. PCR amplification was carried out in a 25 μL reaction mixture containing 12.5 μL of Phusion Hot Start Flex 2 × Master Mix, 2.5 μL of each forward and reverse primer, 50 ng of template DNA, and ddH_2_O to adjust the final volume. The thermal cycling conditions were as follows: initial denaturation at 95 °C for 30 s, followed by 32 cycles of denaturation at 95 °C for 10 s, annealing at 52 °C for 30 s, and extension at 72 °C for 45 s. The PCR products were purified using AMPure XT Beads (Beckman Coulter Genomics, Danvers, MA, USA) and quantified again using the Qubit system. The quality and concentration of the purified libraries were assessed with an Agilent 2100 Bioanalyzer (Agilent Technologies, Santa Clara, CA, USA). Paired-end sequencing (2 × 250 bp) was performed on an Illumina NovaSeq 6000 platform. All amplification and high-throughput sequencing procedures were conducted by LC-Bio Technology Co., Ltd. (Hangzhou, China).

### 2.3. Bioinformatics and Statistical Analysis

Raw paired-end sequencing reads were first demultiplexed based on their barcode sequences, followed by adapter and barcode trimming. Quality filtering, denoising, and Amplicon Sequence Variant (ASV) inference were performed using the DADA2 algorithm [32] via the qiime dada2 denoise-paired command in QIIME 2 (version 2022.2) [33]. Bacterial 16S sequences were taxonomically classified against the SILVA reference database (release 138), and fungal ITS sequences were classified against the UNITE dynamic database (version 8.2), both at a confidence threshold of 0.7. To ensure data quality, singleton ASVs (those with a total count of 1 across all samples) were removed. Furthermore, ASVs classified as non-microbial origin (e.g., host plant sequences), chloroplasts, or mitochondria were also filtered out. The generation of the final ASV abundance table and subsequent functional predictions (bacterial metabolic pathways via PICRUSt2 with KEGG [34], bacterial phenotypes via BugBase [35], and fungal functional guilds via FUNGuild [36]) were performed using the OmicStudio cloud platform (https://www.omicstudio.cn/tool, accessed on 2 March 2026). All other advanced bioinformatic and statistical analyses were implemented in Python (version 3.12.10) within the Visual Studio Code (version 1.131.0) environment. This included the calculation of alpha diversity (Chao1 and Shannon indices) and beta diversity (PCoA based on Hellinger distances; multi-factor PERMANOVA was used to partition community variation, complemented by PERMDISP), the quantification of microbial inheritance and recruitment via Source Tracker with plant tissues from agar-grown plantlets defined as the primary candidate source under the controlled microbe-limited system, and the identification of the Core Microbiome (occupancy ≥ 80%, relative abundance > 0.01%). Differential abundance analysis was performed using LEfSe (LDA > 3.0, *p* < 0.05).

## 3. Results

### 3.1. Cultivation System and Tissue Niche Drive the Diversity and Niche-Specific Dispersal of Grapevine HDEs in a Closed-Subculture System

**Figure 2:**
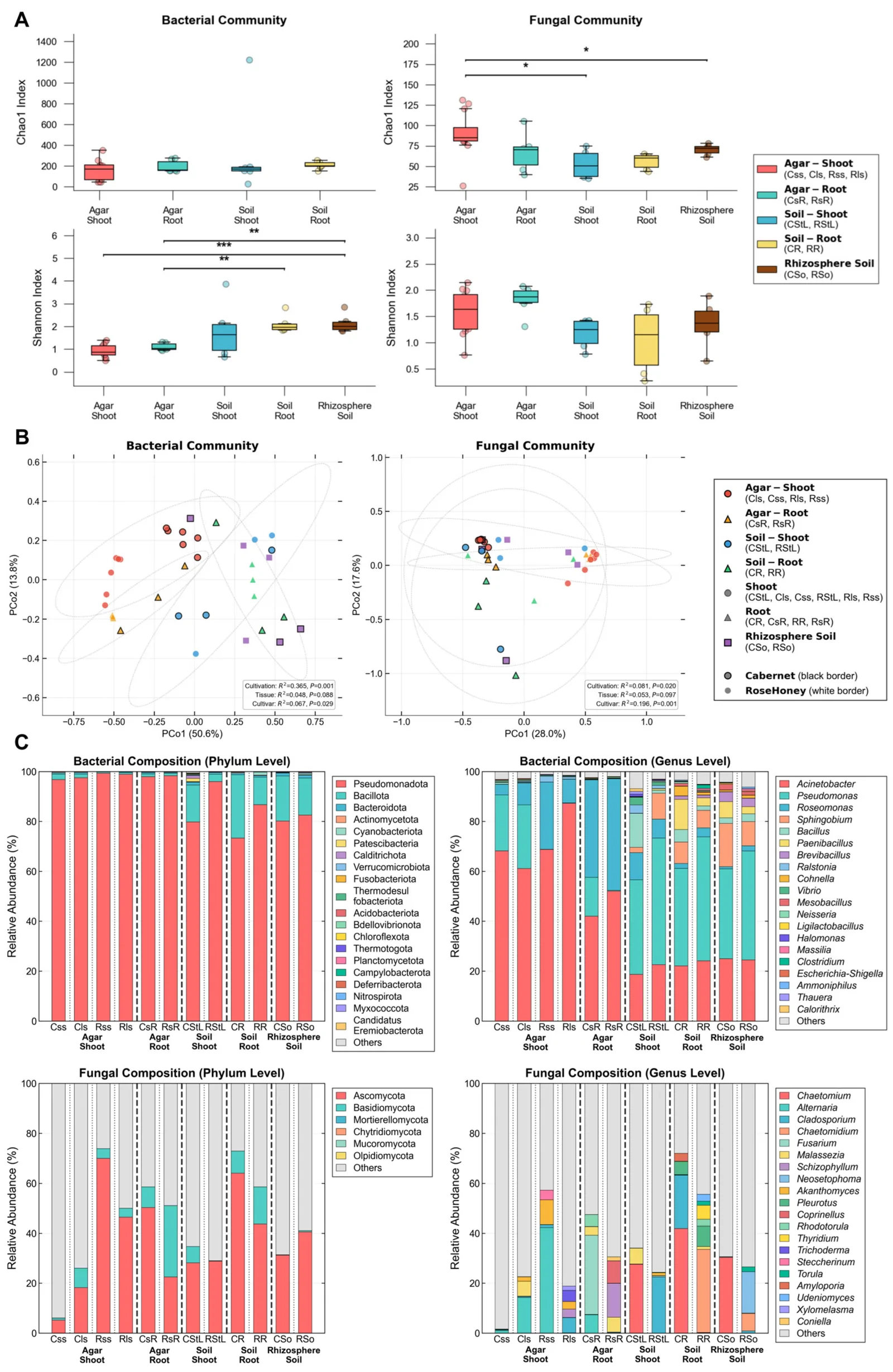
Diversity and niche-specific dispersal of grapevine HDEs in a closed-cultivation system. (**A**) Alpha diversity (Chao1 and Shannon indices) across cultivation systems and tissues for both bacteria and fungi. Boxplots display medians and interquartile ranges; significance determined by Mann–Whitney U tests with Benjamini–Hochberg FDR correction (* *p* < 0.05, ** *p* < 0.01, *** *p* < 0.001). (**B**) Principal coordinate analysis (PCoA) of bacterial and fungal communities based on Hellinger distances. Symbol shapes denote tissues (circle, shoot; triangle, root) and border colors indicate cultivars (black, Cabernet Sauvignon; white, Rose Honey). Ellipses represent 95% confidence intervals; PERMANOVA statistics for cultivation, tissue, and cultivar effects are indicated. (**C**) Relative abundance of dominant bacterial and fungal taxa at the phylum and genus levels. Dashed vertical lines separate sample groups by cultivation system and tissue as defined in (**A**).

**Figure A1:**
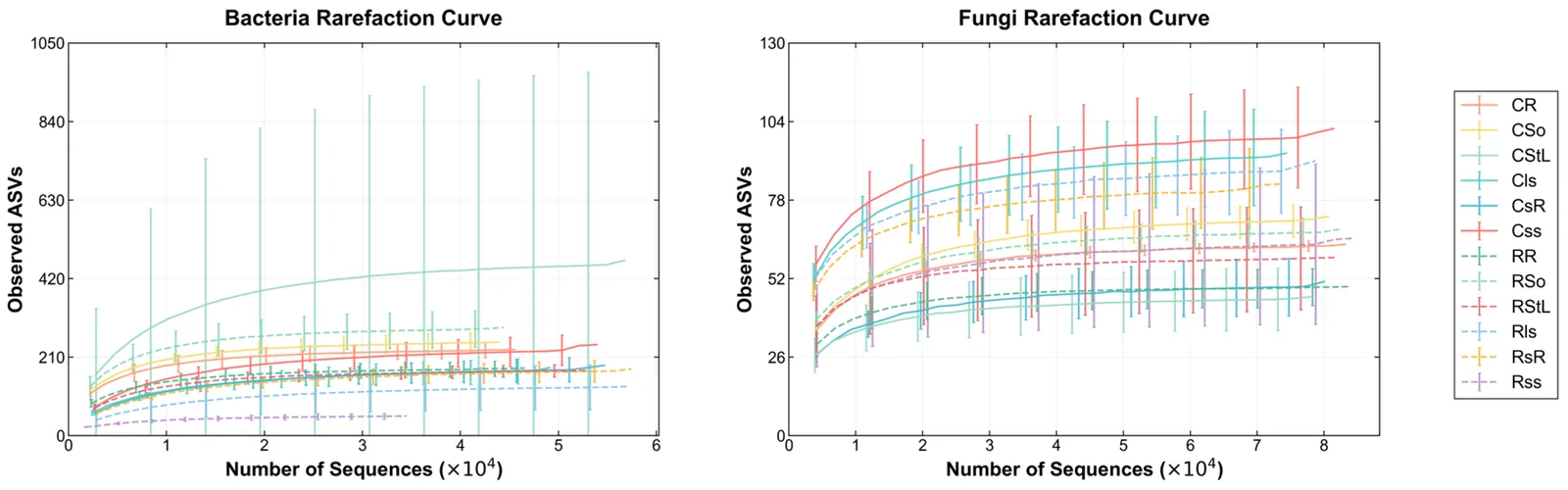
Rarefaction curves of bacterial and fungal communities. (**A**) Bacterial rarefaction curves. (**B**) Fungal rarefaction curves. Solid lines represent observed ASVs as a function of sequencing depth, with shaded areas indicating 95% confidence intervals.

Analysis of the grapevine endophytic microbiota across cultivation systems (agar vs. soil) and tissues (roots vs. shoots) revealed distinct community patterns (Figure 2), with sequencing depth verified as adequate by rarefaction curves (Figure A1). Alpha diversity responses to soil transfer diverged between bacteria and fungi. In bacterial communities, Shannon diversity showed significant pairwise differences after FDR correction, with rhizosphere soil exhibiting higher values than both agar-Shoot (*p* < 0.001) and agar-Root (*p* = 0.006), and soil–Root higher than agar-Root (*p* = 0.006). In fungal communities, Chao1 richness was higher in agar-Shoot than soil–Shoot (*p* = 0.019) and rhizosphere soil (*p* = 0.019) (Figure 2A).

**Figure A2:**
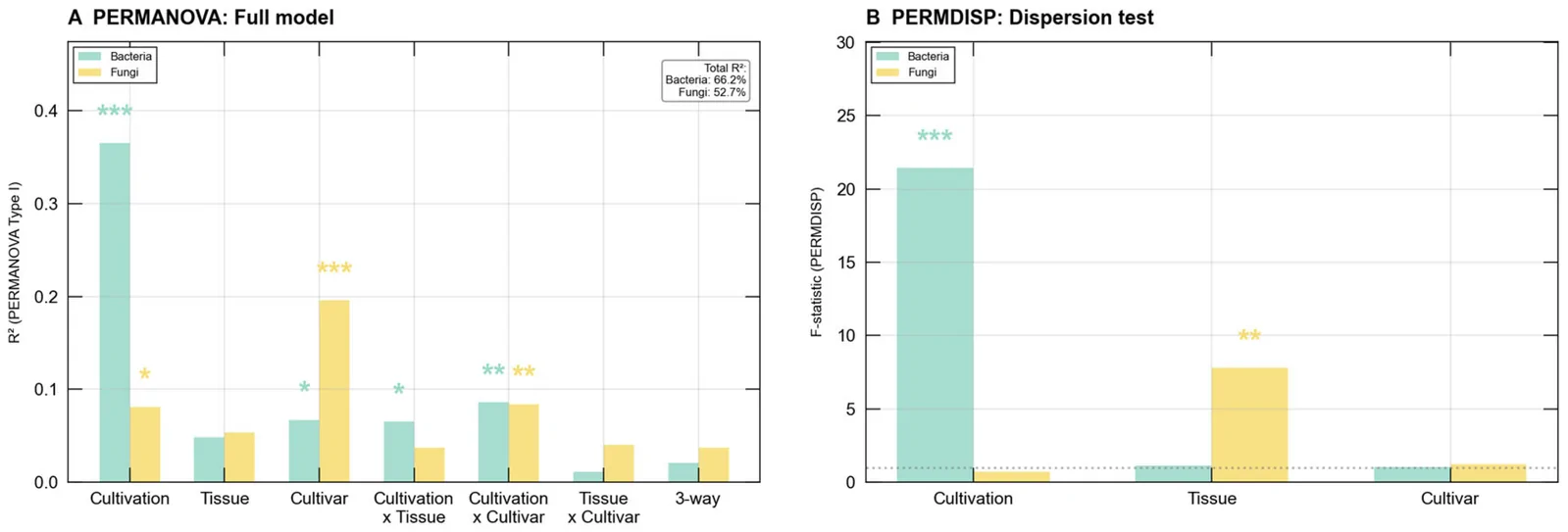
PERMANOVA and PERMDISP results for bacterial and fungal communities. (**A**) Multifactor PERMANOVA based on Hellinger distances, showing the proportion of variance explained (R^2^, Type I) by cultivation, tissue, cultivar, and their interactions. (**B**) PERMDISP results showing F-statistics for multivariate dispersion homogeneity. The dotted horizontal line denotes the null expectation (F = 1). All tests were based on 999 permutations (* *p* < 0.05; ** *p* < 0.01; *** *p* < 0.001).

PCoA based on Hellinger distances showed that cultivation condition was the primary determinant of community structure, with PCo1 explaining 50.6% of the variance for bacteria and 28.0% for fungi. PERMANOVA identified cultivation as the primary driver of bacterial communities (R^2^ = 0.365, *p* = 0.001) and cultivar as the primary driver of fungal communities (R^2^ = 0.196, *p* = 0.001), with tissue compartment showing smaller effects in both kingdoms (bacteria: R^2^ = 0.048, *p* = 0.088; fungi: R^2^ = 0.053, *p* = 0.097) (Figure 2B). Significant two-way and three-way interactions were detected between cultivation, tissue, and cultivar in both bacterial and fungal communities, with total explained variance reaching 66.2% and 52.7%, respectively (Figure A2). Soil-cultivated samples (soil–Shoot, soil–Root, and rhizosphere soil) formed a distinct cluster separate from all agar-grown samples (agar-Shoot and agar-Root). In contrast, fungal communities exhibited greater structural overlap; while habitat remained a significant factor, group separation was less distinct, and the tissue effect was less pronounced (*p* = 0.097) (Figure 2B).

Microbial communities were dominated by core phyla (Figure 2C). Pseudomonadota overwhelmingly dominated bacterial communities (90.65%), followed by Bacillota (7.81%), Bacteroidota, and Actinomycetota, while Ascomycota prevailed among fungi, with Basidiomycota subdominant. Genus-level compositions exhibited habitat-specificity. *Pseudomonas* enriched soil-associated compartments (soil–Root: 44.40%; soil–Shoot: 44.34%; rhizosphere soil: 39.84%) relative to agar-cultivated counterparts (agar-Shoot: 12.04%; agar-Root: 7.94%). Conversely, *Acinetobacter* dominated agar niches (agar-Shoot: 71.36%; agar-Root: 47.07%), while *Roseomonas* peaked in agar-Root (41.89%). *Bacillus* favored soil-cultivated shoots (soil–Shoot vs. agar-Shoot: 7.39% vs. 0.25%). Fungal genera displayed analogous compartment-specificity. *Chaetomium* enriched soil habitats (soil–Root: 20.97%; rhizosphere soil: 15.22%; soil–Shoot: 13.81%), whereas *Alternaria* dominated in agar-Shoot (14.41%) and *Fusarium* in agar-Root (15.92%). *Cladosporium* preferred soil-cultivated tissues (soil–Shoot: 11.27%; soil–Root: 10.73%) (Figure 2C). Community structure remained consistent between cultivars, suggesting strong selection by environmental and tissue-level factors.

### 3.2. Impact of Subculture Pathways on Microbial Inheritance

**Figure 3:**
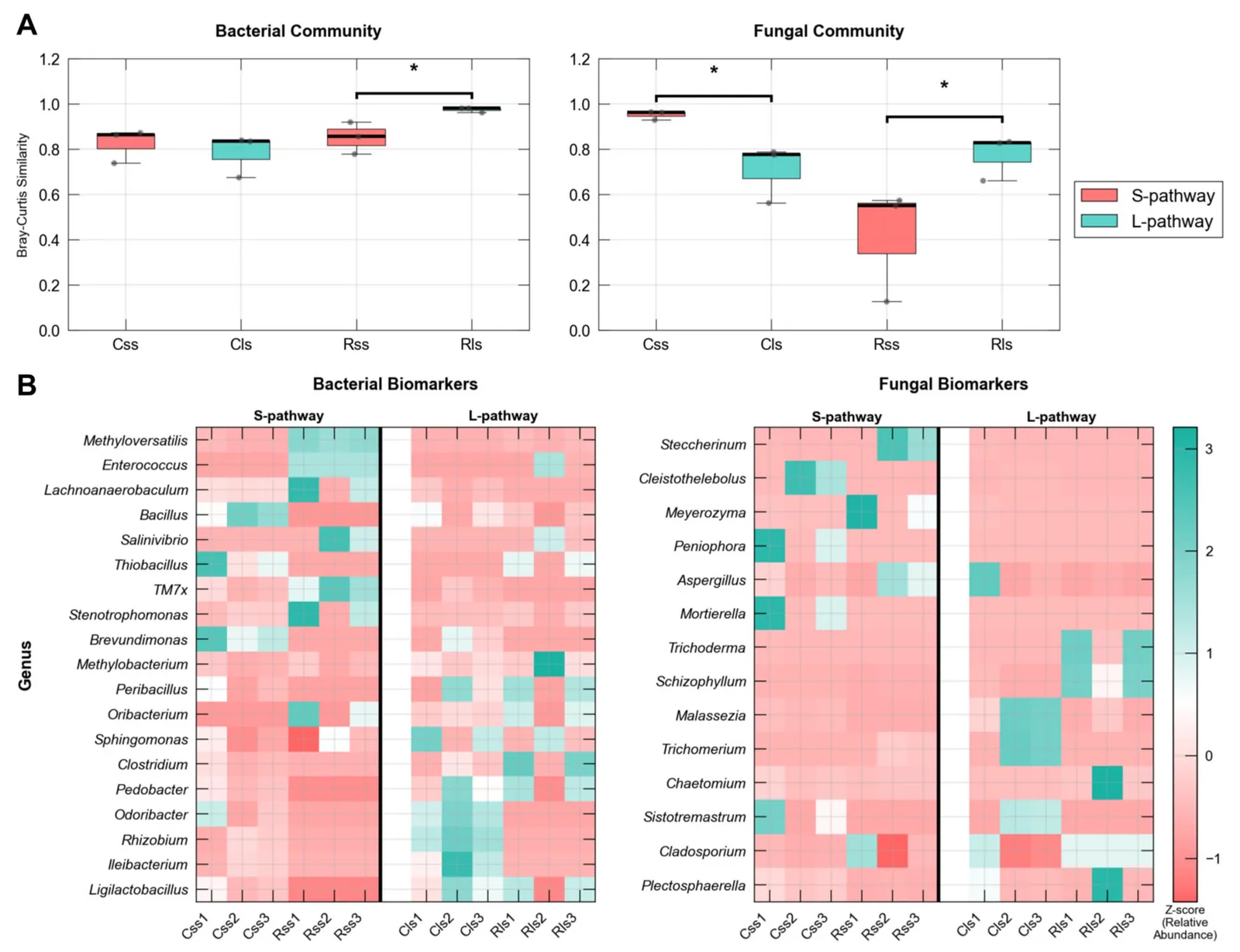
Community similarity and biomarkers across subculture pathways. (**A**) Intra-group Bray–Curtis similarity (based on TSS-normalized ASV data) for bacterial and fungal communities in the S-pathway and L-pathway. Statistical comparisons were performed using the Wilcoxon rank-sum test per cultivar. Asterisks indicate *p* < 0.05. (**B**) Heatmap of row-standardized relative abundance (Z-score) for pathway-enriched taxa. Columns represent individual replicates.

To evaluate the influence of the initial tissue source, endophytic communities from the shoot tip-derived (S) and base-derived (L) pathways were compared over successive subcultures. Analysis of intra-group Bray–Curtis similarity, calculated from pairwise similarities among biological replicates for each cultivar and pathway combination, revealed that the subculture pathway shaped community homogeneity in a host-genotype-dependent manner (Figure 3A). Specifically, in ‘Rose Honey’, the bacterial community in the L-pathway showed significantly higher intra-group similarity than that in the S-pathway (Wilcoxon rank-sum test, *p* < 0.05), which reflected greater compositional uniformity. In contrast, bacterial communities of ‘Cabernet Sauvignon’ exhibited comparable stability in both pathways. Fungal community homogeneity displayed a cultivar-specific pattern that diverged from bacterial trends; while both kingdoms showed higher homogeneity in the L-pathway for ‘Rose Honey’, ‘Cabernet Sauvignon’ exhibited higher fungal homogeneity in the S-pathway (*p* < 0.05). These results partially reflect cultivar-dependent differences in endophyte transmission rates during host growth in agar-grown grapevine plantlets.

**Figure A3:**
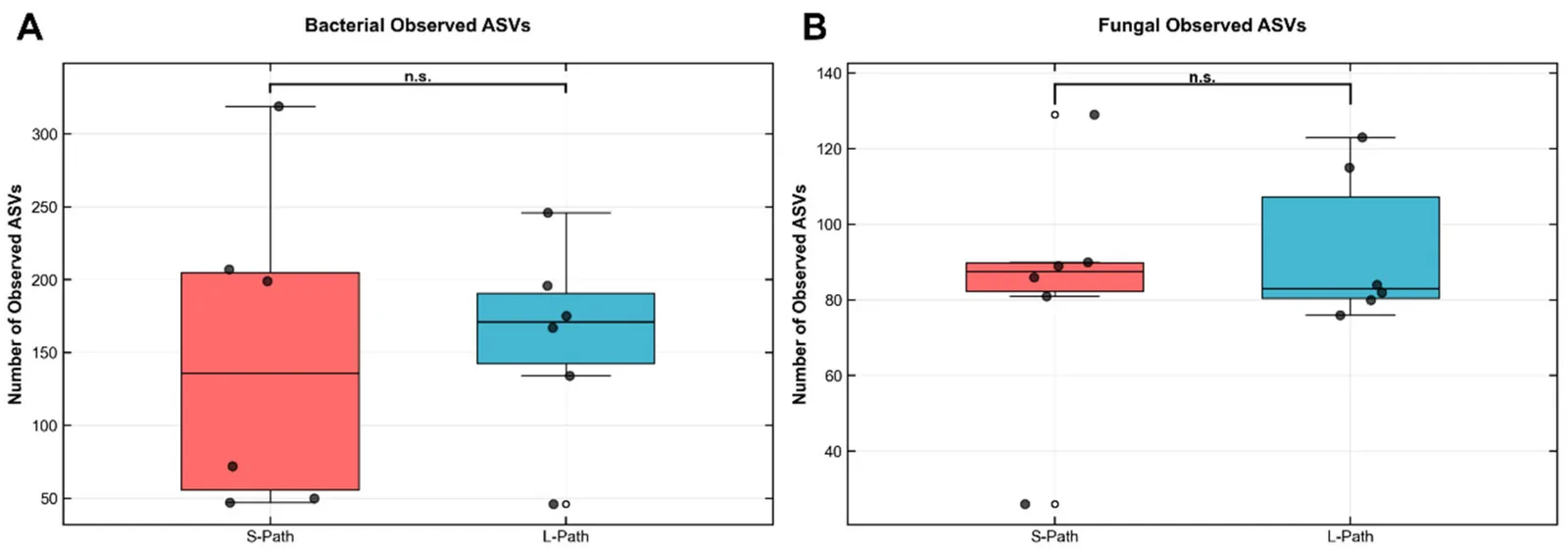
Comparison of observed ASVs between S-Pathway and L-Pathway. (**A**) Bacterial observed ASVs. (**B**) Fungal observed ASVs. Observed ASVs were calculated after rarefaction to equal sequencing depth. Statistical significance was assessed using Mann–Whitney U test (n.s., not significant, *p* > 0.05).

Biomarker analysis identified distinct taxonomic profiles for each pathway (Figure 3B). For the S-pathway, fungal taxa such as *Meyerozyma*, *Steccherinum*, and *Mortierella* showed higher abundance, while the L-pathway was associated with fungal taxa including *Trichoderma*, *Malassezia*, and *Schizophyllum*, along with bacterial taxa such as *Methylobacterium*. Although these taxonomic markers did not reach statistical significance after multiple testing correction, these preliminary observations suggest pathway-specific community differentiation, without any difference in ASV richness between pathways (Figure A3). These results demonstrate that the initial explant source establishes distinct microbial assemblages that persist across subcultures, underscoring the influence of tissue origin on endophyte structure under in vitro conditions.

### 3.3. Outward Migration of Grapevine HDEs During Habitat Transition

**Figure 4:**
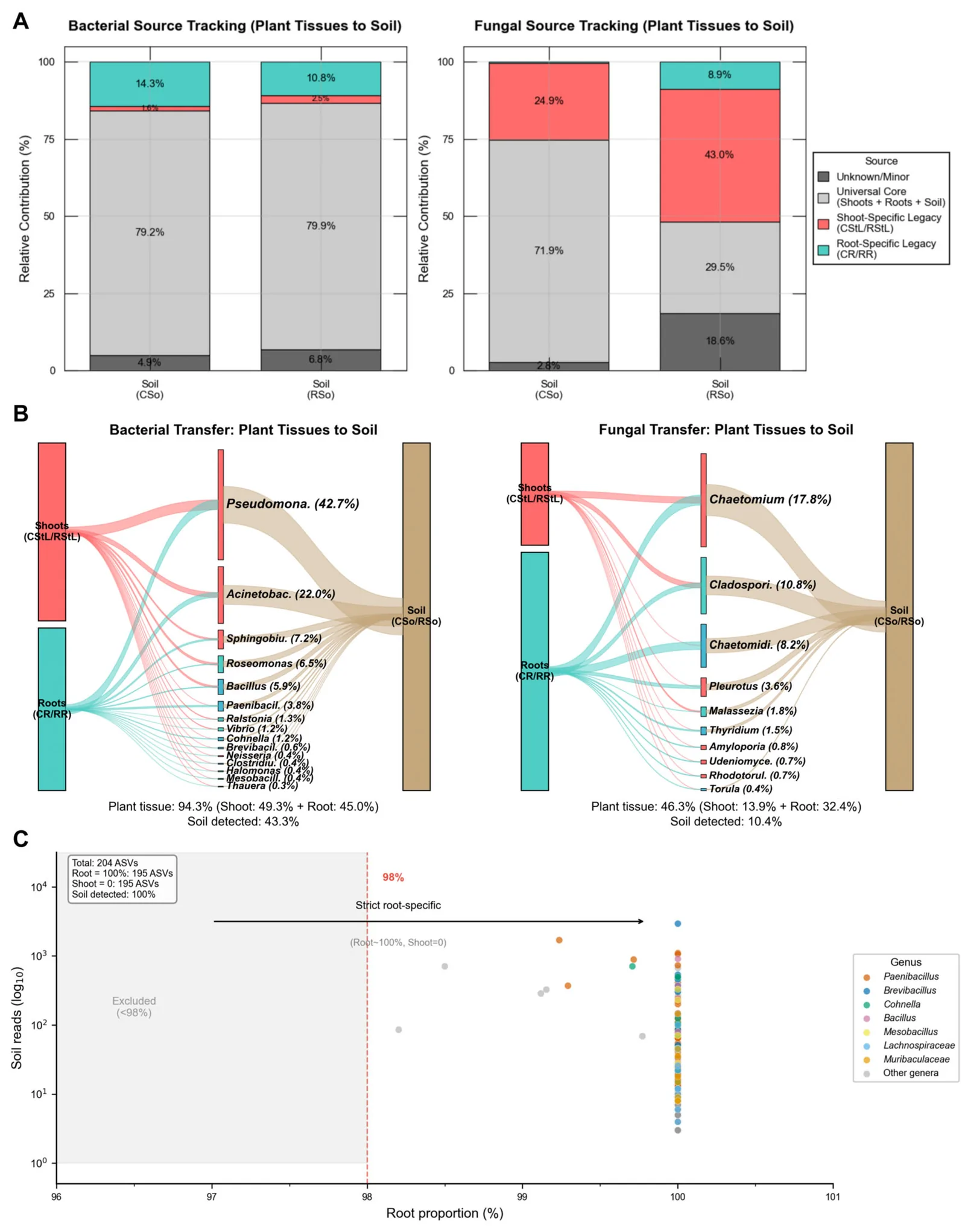
Outward migration of vine HDEs from plantlet to soil. (**A**) Proportional contributions of four source categories to the microbiota in presterilized soil. The Universal Core comprises taxa present in both shoots and roots (30–70% per tissue), whereas Shoot-Specific Legacy and Root-Specific Legacy denote taxa predominantly derived from shoots or roots (>70% in respective tissue), respectively. (**B**) Sankey diagrams showing flux of key bacterial (Top 15) and fungal (Top 10) genera from source compartments (Shoots, Roots) to the soil sink. All percentages are normalized to total plant tissue reads (genus reads in each compartment/total tissue reads × 100%). Soil detected values represent the cumulative soil abundance of displayed genera as a proportion of the total tissue microbiome. (**C**) Root proportion and soil detection of root-specific bacterial ASVs. Root-specific ASVs were defined as ASVs with >98% proportion in roots, detectable in soil, and negligible presence in shoots (*n* = 204 ASVs).

To quantify microbial dispersal following transfer to pre-sterilized soil, source tracking and Sankey flux analyses were performed. Under these microbe-limited conditions, the plantlet served as the major potential source of soil-associated microbiota. Source tracking analysis revealed that the microbial community in the rhizosphere soil was primarily composed of ASVs shared with the plant holobiont, though the contribution of specific tissue niches differed between bacteria and fungi (Figure 4A). For bacteria, the soil community was dominated by a “Universal Core” of taxa present in both shoots and roots, constituting 79.2–79.9% of the community, while root-specific legacy taxa made a significant secondary contribution (10.8–14.3%) compared to the minimal input from shoot-specific lineages (1.6–2.5%). In contrast, fungal distribution showed greater heterogeneity and a higher reliance on tissue-specific migrants. While the Universal Core was the primary source in CSo (71.9%), both Shoot-Specific Legacy (43.0%) and Universal Core (29.5%) contributed substantially to RSo, indicating more compartmentalized source contributions in fungi compared to bacteria and distinct redistribution patterns between soil compartments.

Further sequence flux analysis estimated the relative contribution of dominant microbial taxa from plant tissues to soil samples (Figure 4B). Bacterial transfer was extensive, with 43.3% of tissue-associated sequences detected in soil, driven largely by generalist genera like *Pseudomonas* (42.7%) and *Acinetobacter* (22.0%). Notably, 204 root-enriched ASVs (root proportion > 98%, shoot < 2%), dominated by *Paenibacillus*, were consistently recovered from soil (Figure 4C). Fungal detection was significantly lower, with only 10.4% of tissue-associated sequences detected in the soil. Although root tissues contributed a higher proportion of the total fungal flux (32.4% of the Top 10 genera) compared to shoots (13.9%), this did not translate into high soil detection rates. Collectively, these results demonstrate that tissue-associated bacterial sequences redistribute to a greater extent into soil than fungal sequences under the controlled cultivation conditions.

### 3.4. Maintenance of Core Microbiomes Across Different Grape Cultivars

**Figure 5:**
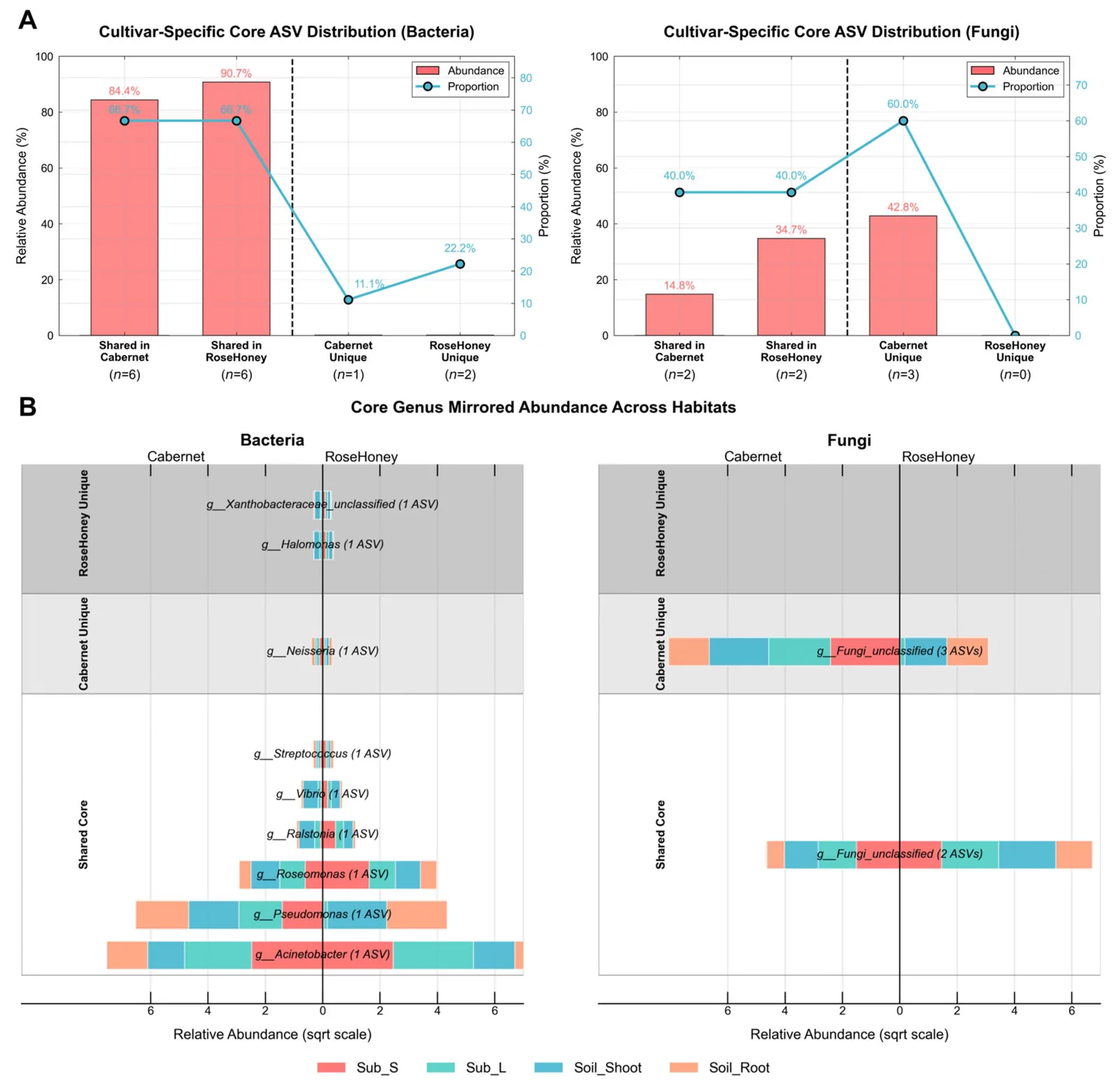
Distribution and persistence of core microbiomes across cultivars. (**A**) Cultivar-specific core ASV distribution. Bars represent relative abundance, and lines indicate the proportion of shared vs. unique core ASVs. (**B**) Mirrored bar plots showing the relative abundance of persistent core bacterial and fungal genera across the four defined habitats (Sub-S, Sub-L, Soil–Shoot, Soil–Root).

The selective effect of host genetics was assessed by identifying the core endophytic ASVs and genera that persisted across all four habitat groups (Sub-S, Sub-L, Soil–Shoot, and Soil–Root). A taxon was included in the core microbiome if it exhibited a prevalence ≥ 80% within each group. The bacterial and fungal core communities exhibited differential conservation between cultivars. The bacterial core was largely conserved, with shared ASVs comprising 84.4% and 90.7% of the total core abundance in ‘Cabernet’ and ‘Rose Honey’, respectively (Figure 5A). In contrast, the fungal core displayed pronounced genotype specificity. Although shared fungal taxa were present (14.8% in ‘Cabernet’ and 34.7% in ‘Rose Honey’), ASVs unique to ‘Cabernet’ contributed substantially (42.8%) to its fungal core abundance, whereas no unique core fungi were identified in ‘Rose Honey’. These results indicate that the bacterial microbiome retains a highly conserved core across genotypes, while the fungal microbiome demonstrates marked host-genotype dependence.

Genus-level analysis confirmed the stability of these core taxa throughout the habitat transition (Figure 5B). Bacterial generalists, including *Acinetobacter*, *Pseudomonas*, and *Roseomonas*, displayed high and consistent presence frequency across all habitats and cultivars, thereby forming a habitat-invariant community backbone. Despite this broad conservation, cultivar-specific signatures were evident. ‘Rose Honey’ uniquely harbored core bacteria such as *Halomonas*, whereas ‘Cabernet’ specifically harbored *Neisseria* among bacteria and showed a higher abundance of unclassified fungal ASVs in the Cabernet-unique category. These persistent core members, being resilient to both environmental and subculture perturbations, may contribute to functional continuity for the host across disparate niches. It is worth noting that the Shoot Specific Legacy taxa observed in Rose Honey (Figure 4A) did not qualify as core members across all four habitats, consistent with the stricter occupancy threshold (≥80% prevalence in each habitat) used for core definition.

### 3.5. Hierarchical Identification and Functional Succession of Core Microbiota

**Figure 6:**
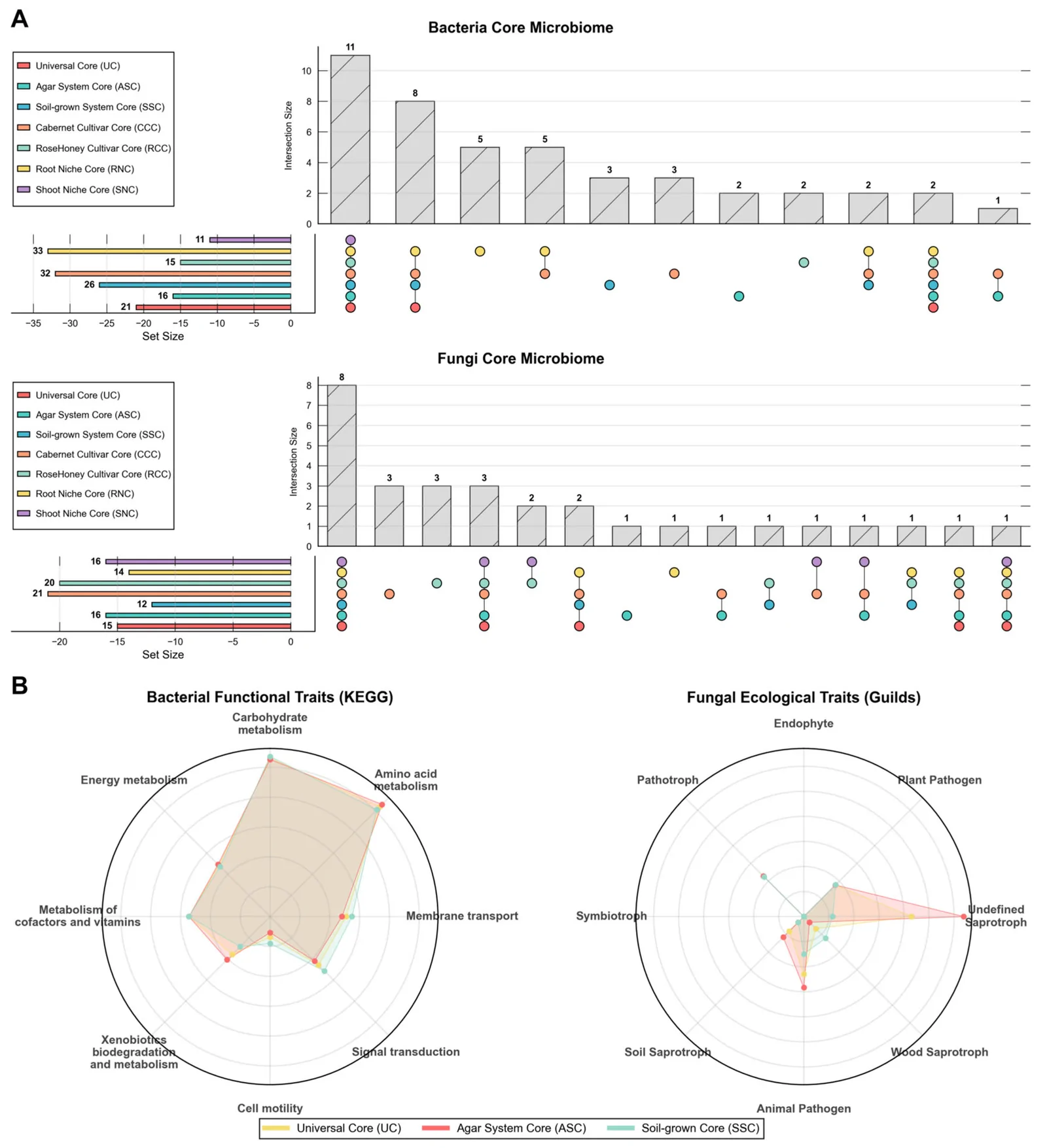
Hierarchical core microbiota and functional niche adaptation. (**A**) Upset plots showing intersections of core ASVs across seven ecological levels, including Universal (UC), System (ASC/SSC), Cultivar (CCC/RCC), and Niche (RNC/SNC) cores. Vertical bars indicate the size of each unique intersection. (**B**) Radar charts of predicted functional profiles for the UC, ASC, and SSC groups. Bacterial functional traits (KEGG level 2) were predicted using PICRUSt2. Fungal ecological traits (guilds) were assigned using the FUNGuild database.

A hierarchical core microbiome, defined across seven analytical partitions, was analyzed to integrate the drivers of the endophytic community and predict functional shifts (Figure 6A). Structural analysis under a stringent occupancy threshold ≥ 80%) revealed a stable backbone of 11 bacterial and 8 fungal ASVs that were present in all seven core sets, constituting a “Universal Core.” Bacterial cores showed pronounced niche specificity, with the root niche core comprising 33 ASVs and the shoot niche core comprising 11 ASVs. The Cabernet cultivar core contained 32 specific bacterial ASVs, whereas the ‘Rose Honey’ cultivar core harbored a comparable bacterial cultivar core (15 ASVs), indicating asymmetric genotype-level selection. Fungal cultivar-specific cores showed a different pattern, with 21 ASVs in Cabernet and 20 ASVs in ‘Rose Honey’. System-specific comparisons revealed that soil cultivation expanded the bacterial core (26 ASVs) but reduced the fungal core (12 ASVs) relative to agar conditions (16 and 16 ASVs, respectively), reflecting differential responses to environmental transitions. Although soil transfer reduced overall bacterial diversity (Figure 2A), the expansion of core ASV richness suggests a convergence towards a more uniformly selected community. Fungal cores exhibited greater combinatorial diversity overall, with more non-empty intersection sets compared to bacteria, reflecting a more flexible colonization strategy.

Functional analysis revealed compartment-associated functional trends (Figure 6B). While core bacterial metabolic functions remained relatively stable across habitats, with conserved carbohydrate and amino acid metabolism dominating all cores, subtle differences were observed in signal transduction pathways (higher in SSC: 6.4% vs. ASC: 5.3%). Fungal adaptation occurred through trophic shifts. Specifically, undefined saprotrophs dominated in the Agar-grown System Core (16.0%), whereas the Soil-grown System Core showed a broader trophic profile that included pathotrophy (5.6%) and plant pathogenicity (4.5%), reflecting increased functional diversity in soil environments.

**Figure A4:**
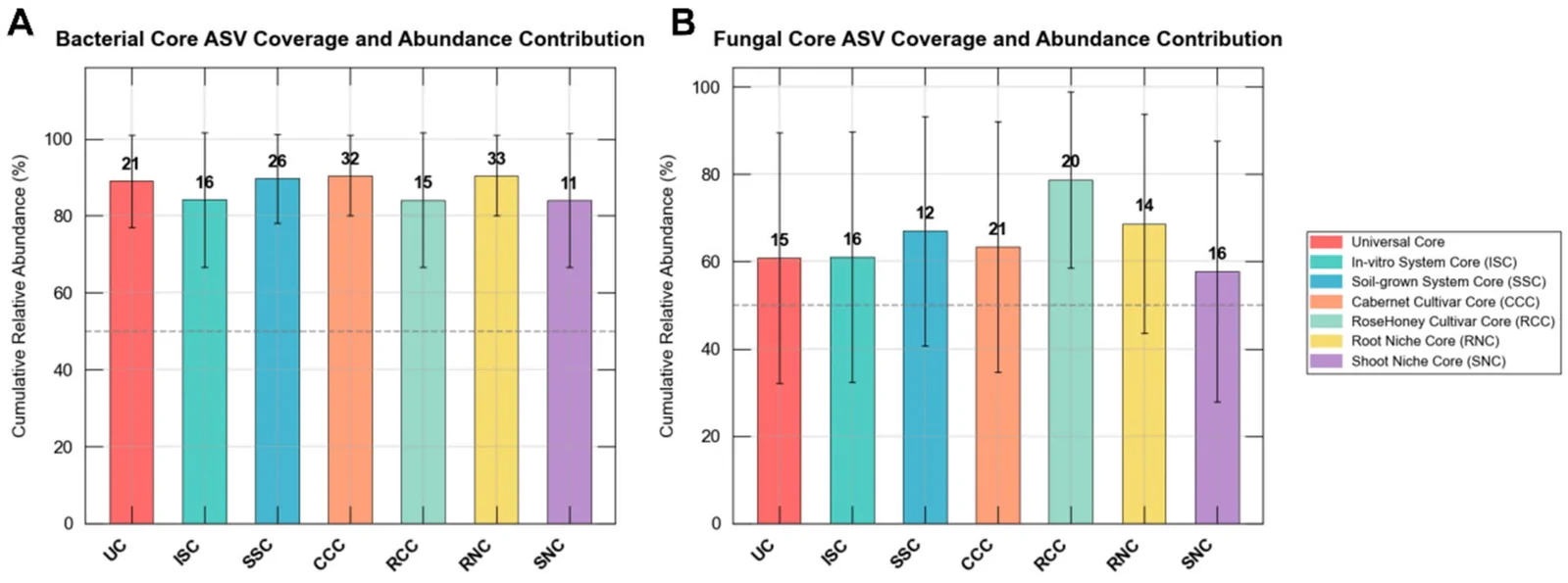
Cumulative relative abundance of core ASVs across hierarchical levels. (**A**) Bacterial core ASVs. (**B**) Fungal core ASVs. Core ASVs were defined at ≥80% prevalence threshold within each hierarchical group.

The core microbiota responds to habitat transitions through differential patterns between bacteria and fungi, whereby bacteria stabilize through conserved metabolic functions, whereas fungi adapt via trophic mode shifts and expanded ecological versatility. Each core set contributed >50% of the abundance in its corresponding group (Figure A4). This hierarchical yet adaptive core ensures the functional continuity of the grapevine endophytic system across different environments.

**Figure 7:**
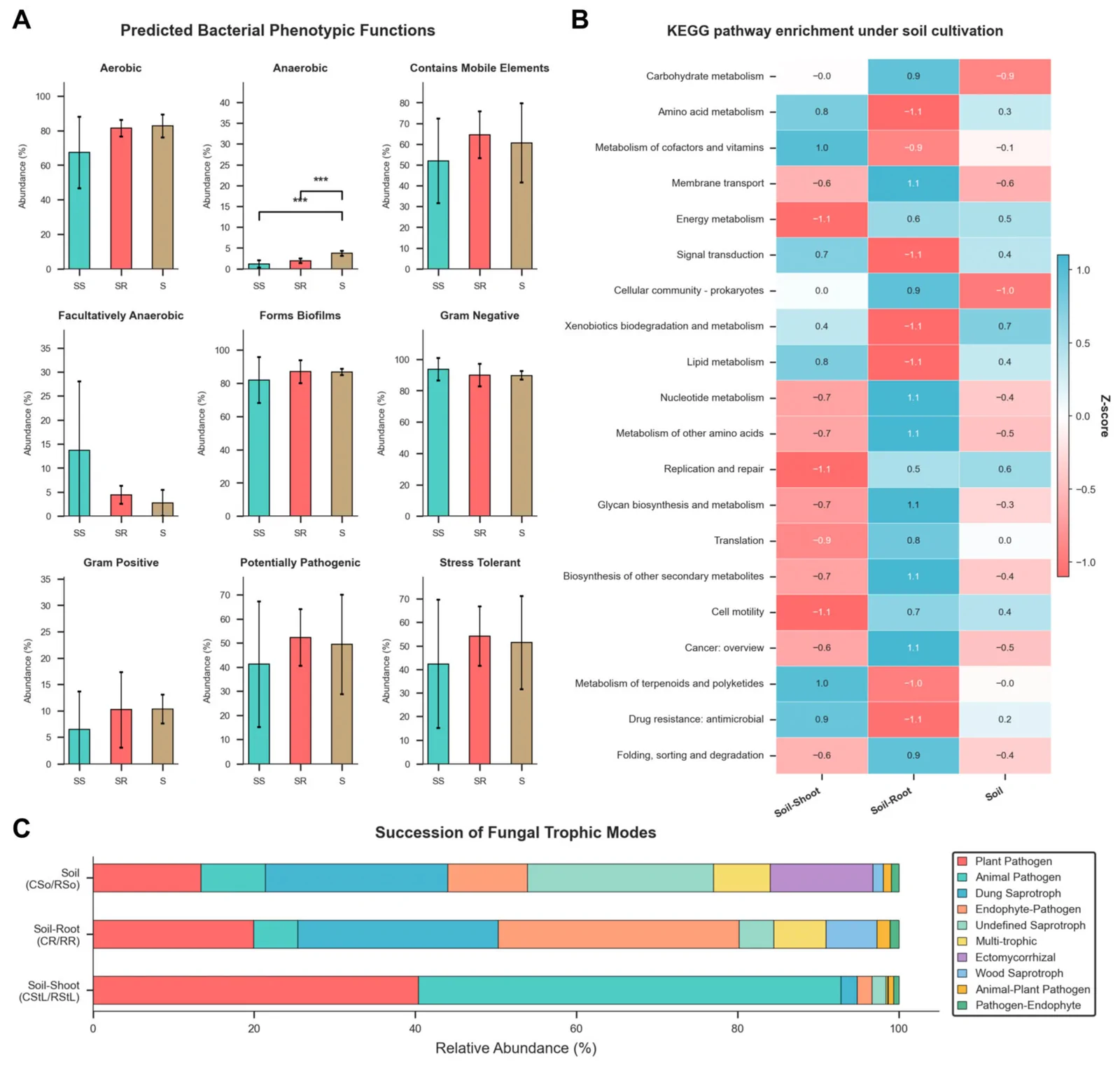
Functional differentiation and ecological assembly of the microbiome during habitat transition. (**A**) Predicted bacterial phenotypic functions via BugBase across three compartments (SS: Soil–Shoot, SR: Soil–Root, S: rhizosphere Soil). Bars represent the mean relative abundance, and error bars indicate the SD. *** *p* < 0.001. (**B**) Heatmap showing the Z-score normalized enrichment of KEGG Level 2 metabolic pathways across the three niches. (**C**) Relative abundance of fungal trophic modes (FUNGuild) across the grapevine-soil system, shown as a stacked bar chart.

To elucidate the potential functional consequences of microbial migration, functional predictions were performed for bacterial and fungal communities across three defined compartments: Soil–Shoot (CStL/RStL), Soil–Root (CR/RR), and rhizosphere Soil (CSo/RSo) (Figure 7). Phenotypic analysis revealed that the anaerobic phenotype was significantly enriched in rhizosphere Soil compared to Soil–Shoot (*p* < 0.001) and Soil–Root (*p* < 0.001). All other tested traits, including aerobic, facultatively anaerobic, stress-tolerant, potentially pathogenic, and mobile elements, remained stable, indicating their conservation across niches (Figure 7A).

KEGG analysis revealed functional partitioning among compartments (Figure 7B). Although no pathway reached statistical significance (*p* > 0.05), the Z-score normalized heatmap revealed distinct functional trends. Soil–Shoot appeared to have relatively higher metabolism of cofactors and vitamins, and metabolism of terpenoids and polyketides. Soil–Root tended to show elevated levels of membrane transport, nucleotide metabolism, glycan biosynthesis, and biosynthesis of secondary metabolites. Rhizosphere Soil displayed relatively higher xenobiotic biodegradation. These results suggest potential metabolic functional differentiation of microbiota across the three niches.

Fungal functional profiles shifted progressively from soil to plant compartments. Taxa annotated as animal pathogens and plant pathogens were most represented in Soil–Shoot (41.1% and 38.2%, respectively). Endophyte-associated complexes and dung saprotrophs showed higher abundance in Soil–Root (27.2% and 21.4%, respectively). Ectomycorrhizal fungi were enriched in rhizosphere Soil (11.7%) (Figure 7C). Notably, ectomycorrhizal fungi were absent from plant compartments, consistent with their introduction into the sterile soil via the plant. This spatial stratification indicates compartment-specific functional partitioning in predicted trophic profiles, with soil favoring saprotrophs and mutualists and host tissues supporting pathogens and endophytes.

## 4. Discussion

### 4.1. Persistence and High Dispersal Efficiency of Host-Dependent Endophytes (HDEs)

This study suggests that HDEs maintain structural stability during in vitro propagation and possess the capacity for habitat expansion upon soil transfer [37]. Our findings support an endogenously driven assembly model wherein the plant holobiont acts as the primary source of the rhizosphere microbiota under conditions of limited environmental input. Within this controlled system, the observed compositional continuity suggests that a subset of HDEs persists during prolonged in vitro propagation and remains detectable after habitat transition. The observed disparity in dispersal efficiency is consistent with known differences in microbial establishment strategies. Bacterial-associated sequences displayed a higher relative contribution to soil communities (43.3%) than fungal-associated sequences (10.4%) after soil transfer. This disparity likely reflects fundamental differences in colonization strategies, wherein the high motility and adaptability of bacteria contrast with the growth constraints and substrate dependency of fungi [38,39]. Early-colonizing bacterial HDEs may facilitate subsequent community assembly by rapidly establishing in newly available niches [40].

Taxonomically, the high relative abundance contribution rates of *Pseudomonas* and *Acinetobacter* corroborate their roles as generalists [41,42]. Their broad metabolic plasticity and efficient motility likely facilitate transitions from restricted endophytic niches to complex soil habitats. This high bacterial dispersal is further supported by source-tracking analysis, which revealed that soil bacterial communities were predominantly sustained by taxa shared between shoots and roots; notably, the Universal Core alone contributed approximately 80% of the total soil bacterial community composition. Conversely, the limited fungal migration efficiency reflects divergent life history strategies and specialized symbiotic requirements, as fungi retained a stronger dependence on tissue-specific legacies, which may restrict their independent dispersal in the absence of a living host substrate.

### 4.2. Spatial Inheritance and Tissue-Specific Imprints of HDEs

The initial anatomical location establishes a persistent spatial signature that influences microbial assembly following repeated subculture. This process is governed by host-driven selection imposed by internal physiological gradients, including variations in oxygen, nutrient availability, and plant hormones [43]. Data indicate that bacterial communities in the L-pathway maintained higher homogeneity compared to the S-pathway, particularly in ‘Rose Honey’. This disparity is consistent with the niche restriction hypothesis, as the more mature and metabolically stable base tissue may present a more consistent niche than the actively growing, meristematic shoot tip [44].

Crucially, cultivar-dependent effects modulate these imprints, with host genotype exerting a more pronounced filtering role on fungal communities than on bacterial ones. Notably, this cultivar effect was pathway-specific; ‘Rose Honey’ exhibited higher bacterial homogeneity via the L-pathway, whereas ‘Cabernet Sauvignon’ showed higher fungal homogeneity via the S-pathway, underscoring interactive effects between host genetics and explant source. While specific biomarkers (e.g., *Methylobacterium* in the L-pathway) require further validation, their enrichment from amplicon sequencing data suggests a structured transmission pattern maintained during agar propagation. This pattern indicates that the initial tissue niche contributes to maintaining compartment-specific microbial assemblages during repeated subculture [45].

### 4.3. Dominance of the Universal Core and Establishment of the Root-Specific Legacy

The assembly of the rhizosphere microbiome was predominantly driven by the internal plant microbiota, suggesting an endogenous successional trajectory. The Universal Core, comprising 11 bacterial and 8 fungal ASVs, served as the structural backbone, contributing approximately 80% of the soil bacterial community. This dominance aligns with the principle of functional redundancy [46,47], wherein conserved metabolic pathways likely ensure community stability despite environmental fluctuations. The greater conservation of bacterial core taxa across the two cultivars than fungi further suggests that bacterial community composition was less affected by host genotype under the present experimental conditions, whereas fungal communities exhibited stronger cultivar specificity. The stability of core bacterial metabolic functions is consistent with these taxa contributing to nutrient cycling and host-associated processes. In addition to the Universal Core, the delineation of the Root-Specific Legacy accounting for 10.8–14.3% of the soil bacterial community likely represents a specialized bacterial reservoir activated by the transition to soil. The recovery of 204 root-enriched ASVs, dominated by *Paenibacillus*, from soil supports this interpretation. The persistence of these root-associated ASVs after soil transfer suggests that root tissues represent an important reservoir of bacterial diversity during habitat transition [48]. Together, the Universal Core and Root-Specific Legacy indicate a hierarchical organization of host-associated bacterial communities, in which conserved taxa provide a stable microbial foundation while niche-associated members contribute additional community variation following environmental change.

### 4.4. Functional Reconfiguration and Ecological Strategy Shifts During Habitat Transition

Functional predictions derived from PICRUSt2, BugBase, and FUNGuild provided insights into the potential ecological roles of HDEs during habitat transition. Conserved metabolic profiles of the bacterial core suggested functional continuity across habitats, while enrichment of membrane transport, nucleotide metabolism, glycan biosynthesis, and secondary metabolite biosynthesis in the Soil–Root community indicated that Root-Specific Legacy taxa may possess metabolic potentials relevant to nutrient exchange, environmental adaptation, and plant–microbe interactions [49,50]. In fungi, the expanded diversity of predicted trophic categories after soil transfer reflected broader ecological differentiation relative to the controlled agar system. Collectively, these findings suggest that bacterial HDEs may rely on conserved and adaptive metabolic potentials, whereas fungal HDEs may respond via ecological strategy shifts. Although these interpretations are based on amplicon-derived predictions, they offer a conceptual framework for further validation through metagenomic, metatranscriptomic, and physiological approaches.

Building on these functional insights, the identification of Universal Core taxa and the Root-Specific Legacy offers insights into how host-associated microbial reservoirs may contribute to plant microbe interactions and provides a basis for exploring microbiome-based strategies in perennial crops. Focusing on two grapevine cultivars within a controlled in vitro-to-soil transition system, this study provides a targeted framework for dissecting microbial persistence and redistribution. Extending this framework to additional genotypes and complex environmental conditions will help assess the broader applicability of these patterns.

## Data Availability

All plant lines and microbial strains are available upon request. All data and materials are available in the manuscript and in NCBI under the accession numbers PRJNA1456428 and PRJNA1456452. All study data are included in the article.

## Abbreviation

The following abbreviations are used in this manuscript:

Css	Cabernet Sauvignon Shoot-tip Shoot
Rss	Rose Honey Shoot-tip Shoot
Cls	Cabernet Sauvignon Lateral-basal Shoot
Rls	Rose Honey Lateral-basal Shoot
CsR	Cabernet Sauvignon Shoot-tip Root
RsR	Rose Honey Shoot-tip Root
CStL	Cabernet Sauvignon Soil Tissue Leaf (shoot)
RStL	Rose Honey Soil Tissue Leaf (shoot)
CR	Cabernet Sauvignon Root
RR	Rose Honey Root
CSo	Cabernet Sauvignon Soil Origin
RSo	Rose Honey Soil Origin
IBA	Indole-3-butyric acid
6-BA	6-benzylaminopurine
